# Nonresective Emergency Management of Perforated Sigmoid Diverticulitis: A Case Report

**DOI:** 10.7759/cureus.105847

**Published:** 2026-03-25

**Authors:** Vijay Naraynsingh, Parvati Raghunath, Dave Harnanan, Yardesh Singh

**Affiliations:** 1 Clinical Surgical Sciences, University of the West Indies at St. Augustine, St. Augustine, TTO; 2 Surgery, Medical Associates Hospital, St. Joseph, TTO; 3 Surgery, University of the West Indies at St. Augustine, St. Augustine, TTO

**Keywords:** diverticular abscess, general and vascular surgery, hartmann’s procedure, hinchey stage iv diverticulitis, intra-abdominal abscess, non-resection in emergency surgery, perforated sigmoid diverticulitis, rate of surgical management of acute diverticulitis, sigmoid diverticulitis, small bowel diverticular disease

## Abstract

This case report aims to demonstrate that it is not always necessary to resect the sigmoid colon in emergency surgery for perforated diverticulitis. We have found that in several cases, most of the sigmoid is in fact quite healthy, and the perforation is limited to a very small area. This is demonstrated by a recent case where a 44-year-old male presented to our care with severe sepsis and extensive peritonitis with abscess formation. At emergency surgery, the abscess was drained, but the abdominal sepsis was too extensive to permit safe resection and primary anastomosis. The sigmoid was severed at the site of perforation, and the distal stump was oversewn and tacked near the colostomy site with no resection of the sigmoid. Four months later, a contrast enema showed that most of the distal sigmoid was normal. It was therefore easy and safe to do colostomy reversal with minimal excision of the margins on each side. In the local/Caribbean setting, we could avoid permanent colostomy by minimizing/avoiding resection of otherwise normal sigmoid colon in perforated diverticulitis. After the sepsis has settled, the colon can be studied to determine how much colon, if any, should be resected at ostomy reversal.

## Introduction

Sigmoid diverticulitis is a common condition encountered by surgeons in the acute setting [1]. When this is complicated by abscess and perforation, it often presents with severe peritonitis and sepsis. The selection of an adequate procedure for management is hampered by the heterogeneity of the literature [2].

Traditionally, management involves resection of the affected colon, either through Hartmann’s procedure or via primary anastomosis, with or without a defunctioning stoma. While effective in controlling infection, these approaches often result in permanent colostomy [3]. In many cases, however, the majority of the sigmoid colon remains healthy, and extensive resection may not be needed.

Herein, we present a case in which the sigmoid colon was preserved during emergency surgery by limiting intervention to the perforated site, allowing definitive resection and colostomy reversal to be safely performed months later. This strategy highlights a tailored approach that can possibly reduce complications and improve quality of life, particularly in settings with limited resources, as in the Caribbean. Furthermore, the aim of this case report is to demonstrate that conservative management of the sigmoid colon during emergency surgery can safely avoid unnecessary resection and permanent colostomy in selected patients for perforated diverticulitis.

## Case presentation

**Figure 1:**
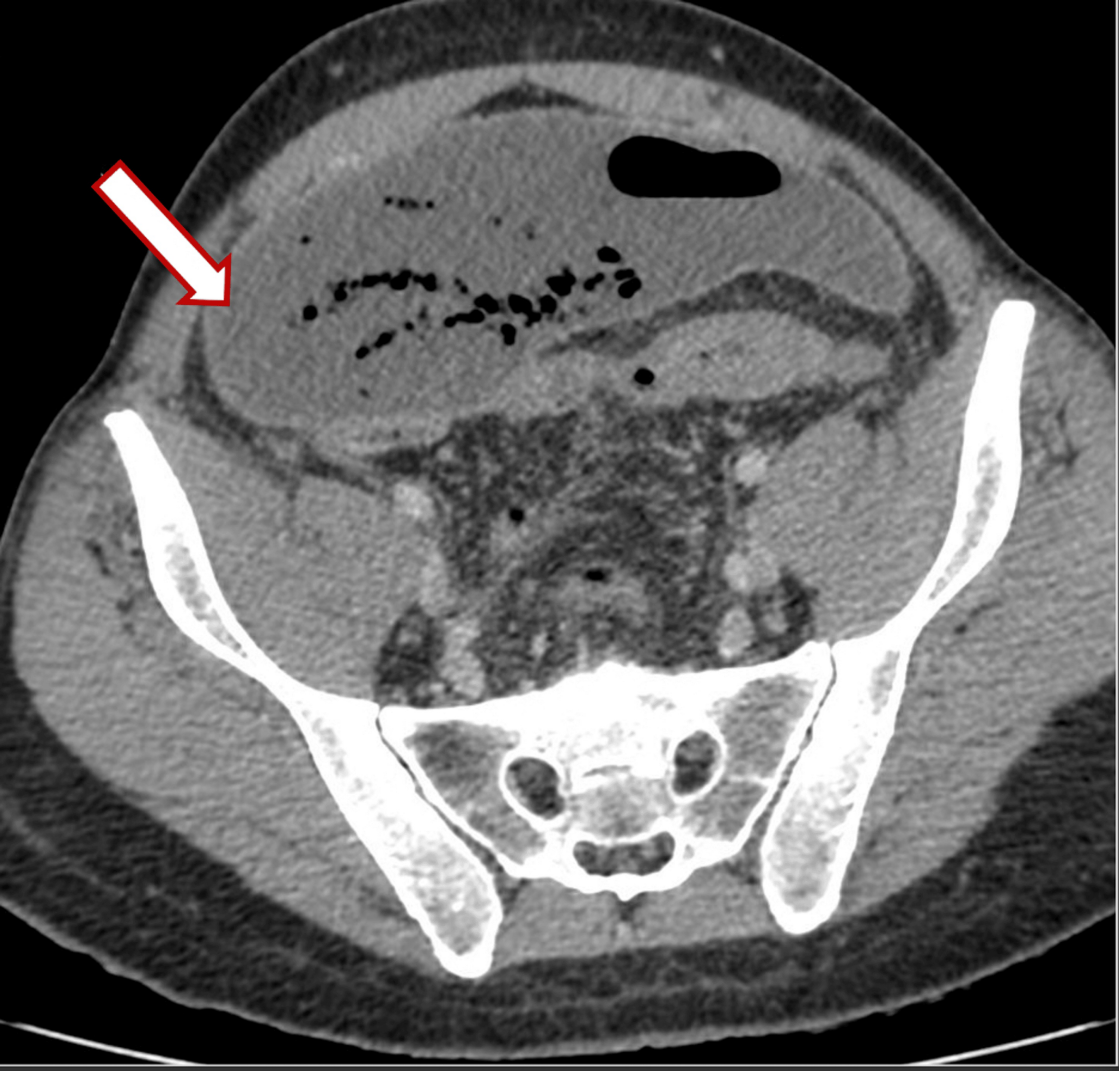
CT imaging demonstrating a large abscess cavity in the lower abdomen containing air and fluid, extending anteriorly to the ventral abdominal wall, with maximal dimensions of 20 cm (TS) × 8 cm (AP) × 19 cm (CC). The collection contacts the distal sigmoid colon, with a few tiny adjacent air locules indicating the site of perforation. The white arrow (outlined in red) indicates the abscess cavity. TS: transverse; AP: anteroposterior; CC: craniocaudal

**Figure 2:**
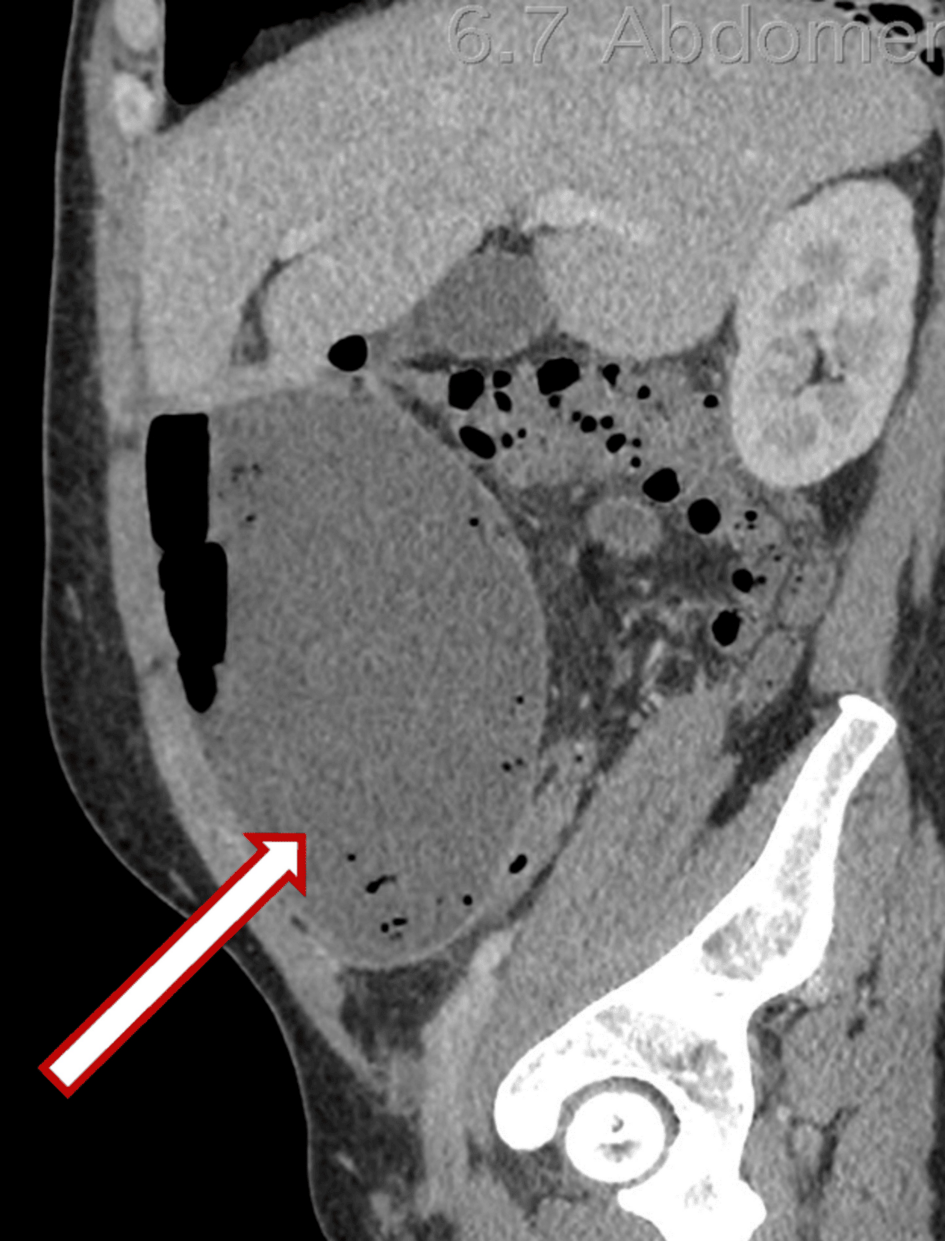
Sagittal view demonstrating a large abscess cavity in the lower abdomen containing air and fluid, extending anteriorly toward the abdominal wall, with maximal dimensions of 20 cm (TS) × 8 cm (AP) × 19 cm (CC). The collection contacts the inferior margin of the right hepatic lobe. The white arrow (outlined in red) indicates the abscess cavity. TS: transverse; AP: anteroposterior; CC: craniocaudal

**Figure 3:**
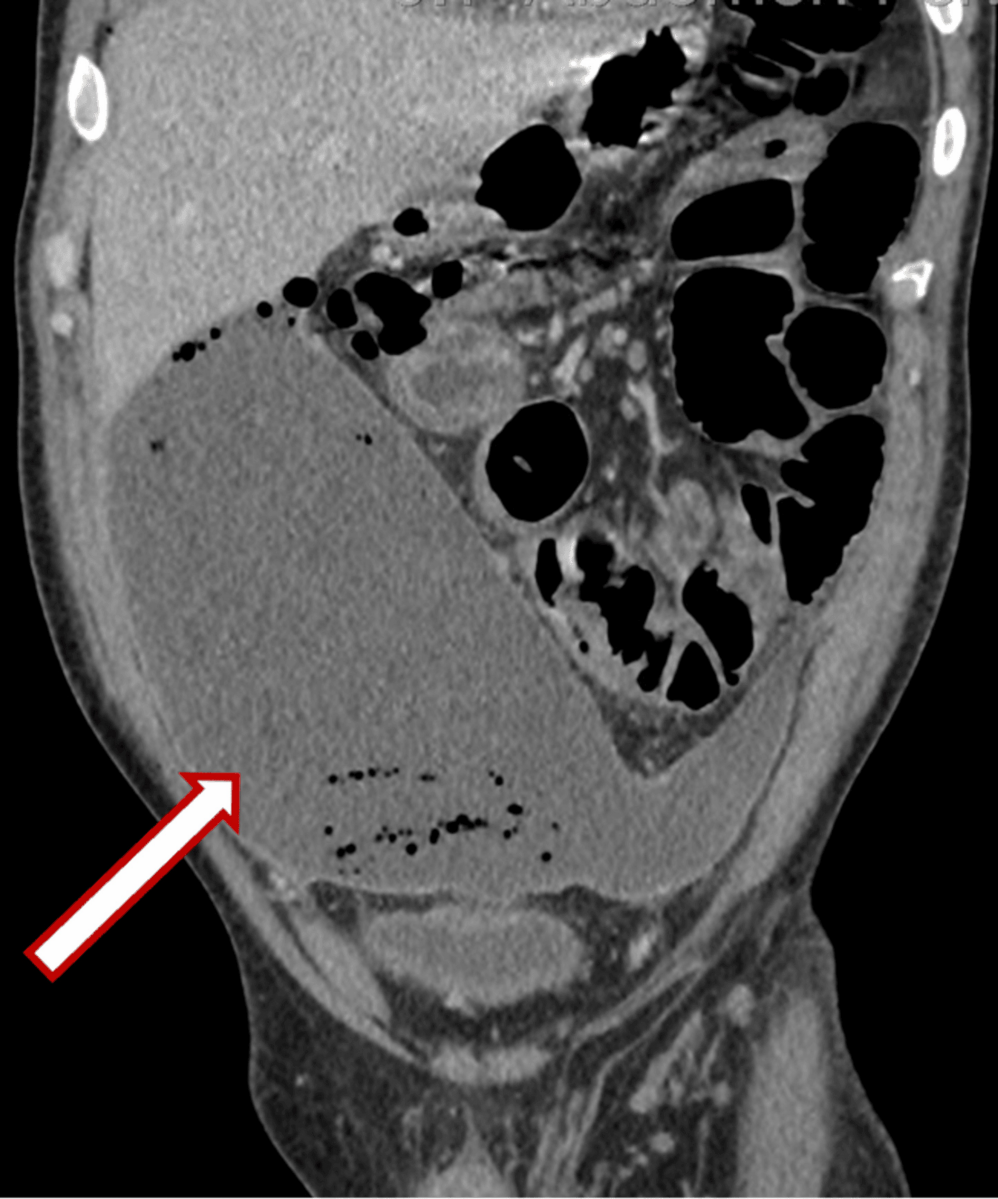
Coronal view demonstrating a large abscess cavity in the lower abdomen containing air and fluid, extending posterior to the urinary bladder, with maximal dimensions of 20 cm (TS) × 8 cm (AP) × 19 cm (CC). The collection contacts the inferior margin of the right hepatic lobe. The white arrow (outlined in red) indicates the abscess cavity. TS: transverse; AP: anteroposterior; CC: craniocaudal

A 44-year-old male presented with a five-day history of severe abdominal pain. The patient appeared to be quite ill-looking and was moderately dehydrated. On examination, the patient had a pulse of 120 beats per minute, a temperature of 38.4°C, a respiratory rate of 24 breaths per minute, and a blood pressure of 100/60 mmHg. He had moderate abdominal distension with generalized tenderness and more marked peritonitis in the lower abdomen. A CT abdomen and pelvis then showed a large abscess cavity across the lower abdomen with air and fluid contents, extending posterior to the urinary bladder and anteriorly to the ventral abdominal wall, with maximal dimensions of 20 cm (transverse, or TS) × 8 cm (anteroposterior, or AP) × 19 cm (craniocaudal, or CC). The collection contacted the inferior margin of the right lobe of the liver (Figures 1-3).

**Figure 4:**
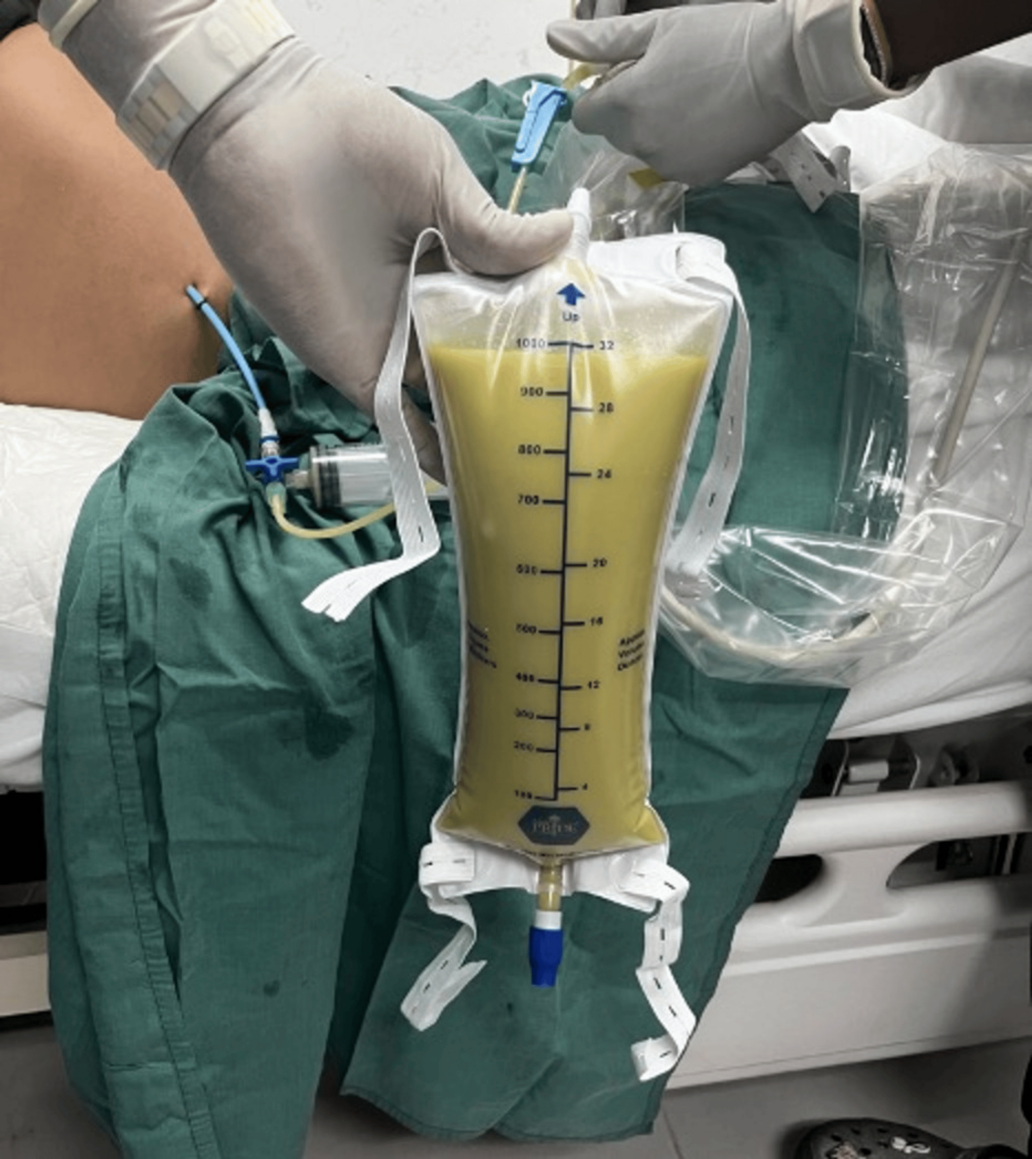
Percutaneous drainage of the initial purulent pus.

The patient was resuscitated with fluids and intravenous antibiotics, and percutaneous drainage of the abscess was performed via interventional radiology, yielding 3 L of thick yellow pus (Figure 4).

**Figure 5:**
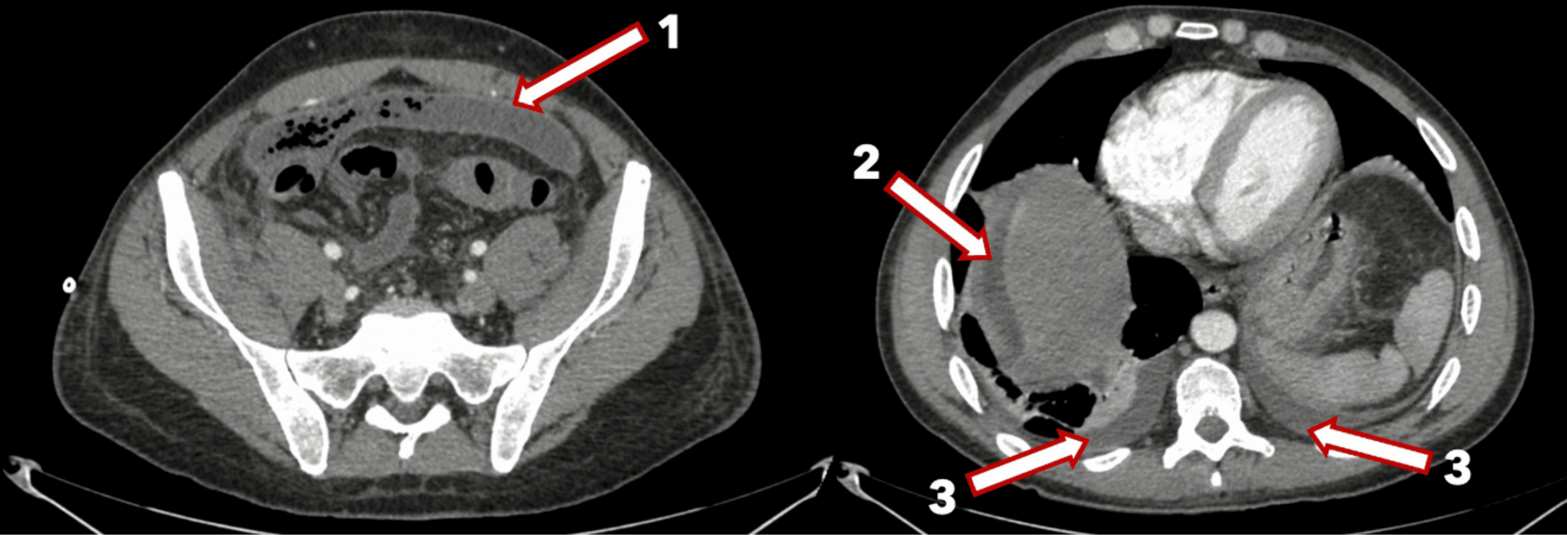
CT abdomen and pelvis demonstrating a reduction in the abscess volume, measuring 5 cm (AP) × 15 cm (TS) (1), with stable right subdiaphragmatic and subhepatic fluid (2) and minimal bilateral pleural effusions (3). White arrows (outlined in red) indicate the described findings. TS: transverse; AP: anteroposterior

After 20 hours of drainage, the patient continued to be febrile and tachycardic, and a repeat CT abdomen and pelvis scan was done, which showed that the abscess was reduced in volume, measuring 5 cm AP x 15 cm TS with stable right subdiaphragmatic fluid and minimal bilateral pleural effusions (Figure 5).

The patient was then taken to surgery, where a midline laparotomy was performed, and thick, foul-smelling pus was suctioned. The peritoneum, gut, and omentum were matted together. A sigmoid perforation was identified along the anteroinferior wall of the distal sigmoid colon. The site of perforation was approximately 18 cm from the anorectal junction, and the gut was transected at this point.

The proximal end was brought out as an end colostomy, and the distal end was oversewn and tacked to the anterior abdominal wall near the colostomy site. Notably, no resection was performed.

**Figure 6:**
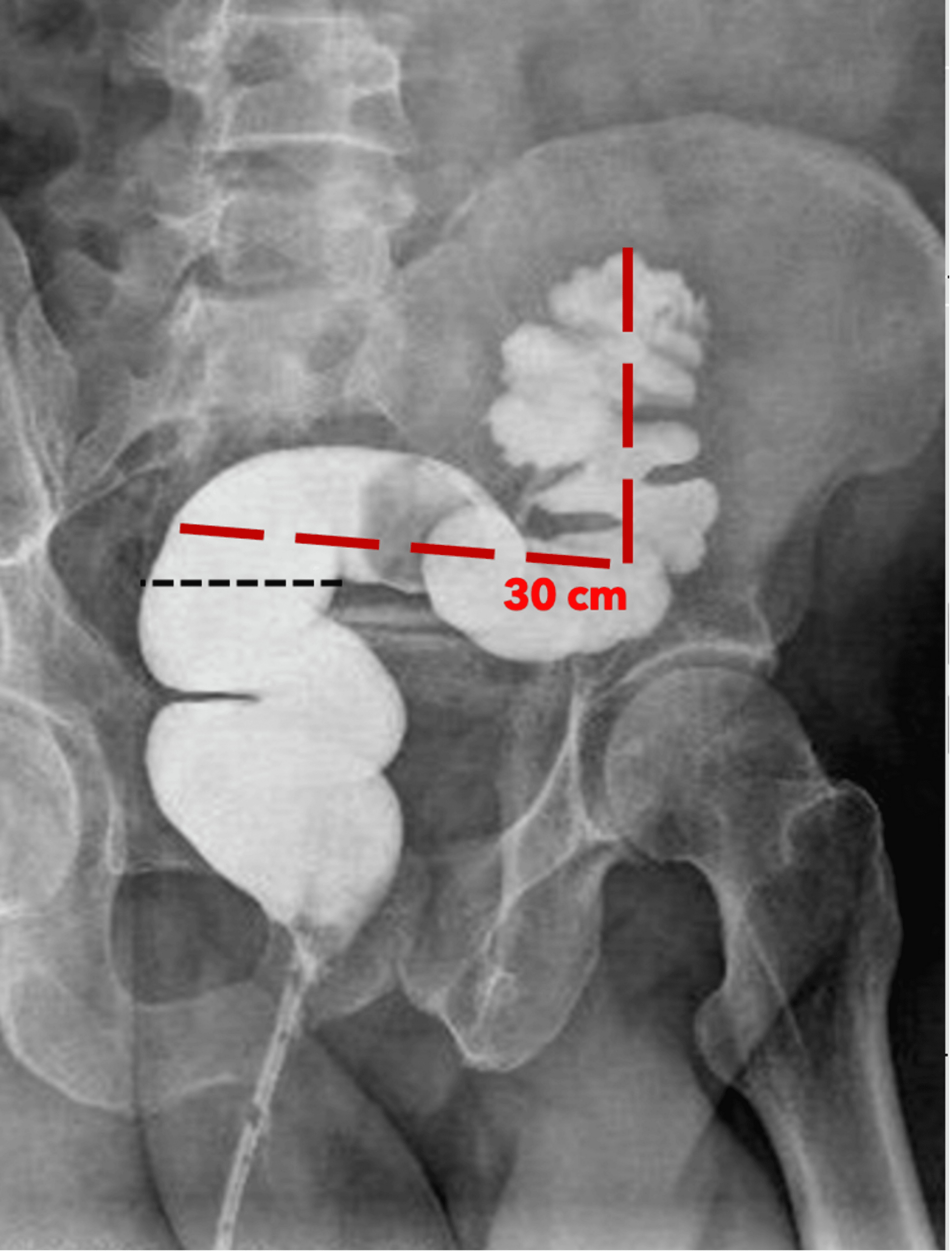
Scan showing 30 cm of healthy sigmoid colon proximal to the rectosigmoid junction. The black dotted line marks the rectosigmoid junction.

The patient was discharged seven days postoperatively and was able to go home on a normal diet. The stoma functioned well with no leakage, and there was no evidence of ongoing sepsis. Four months later, a contrast study of the distal colon showed 30 cm of healthy sigmoid colon proximal to the rectosigmoid junction (Figure 6).

**Figure 7:**
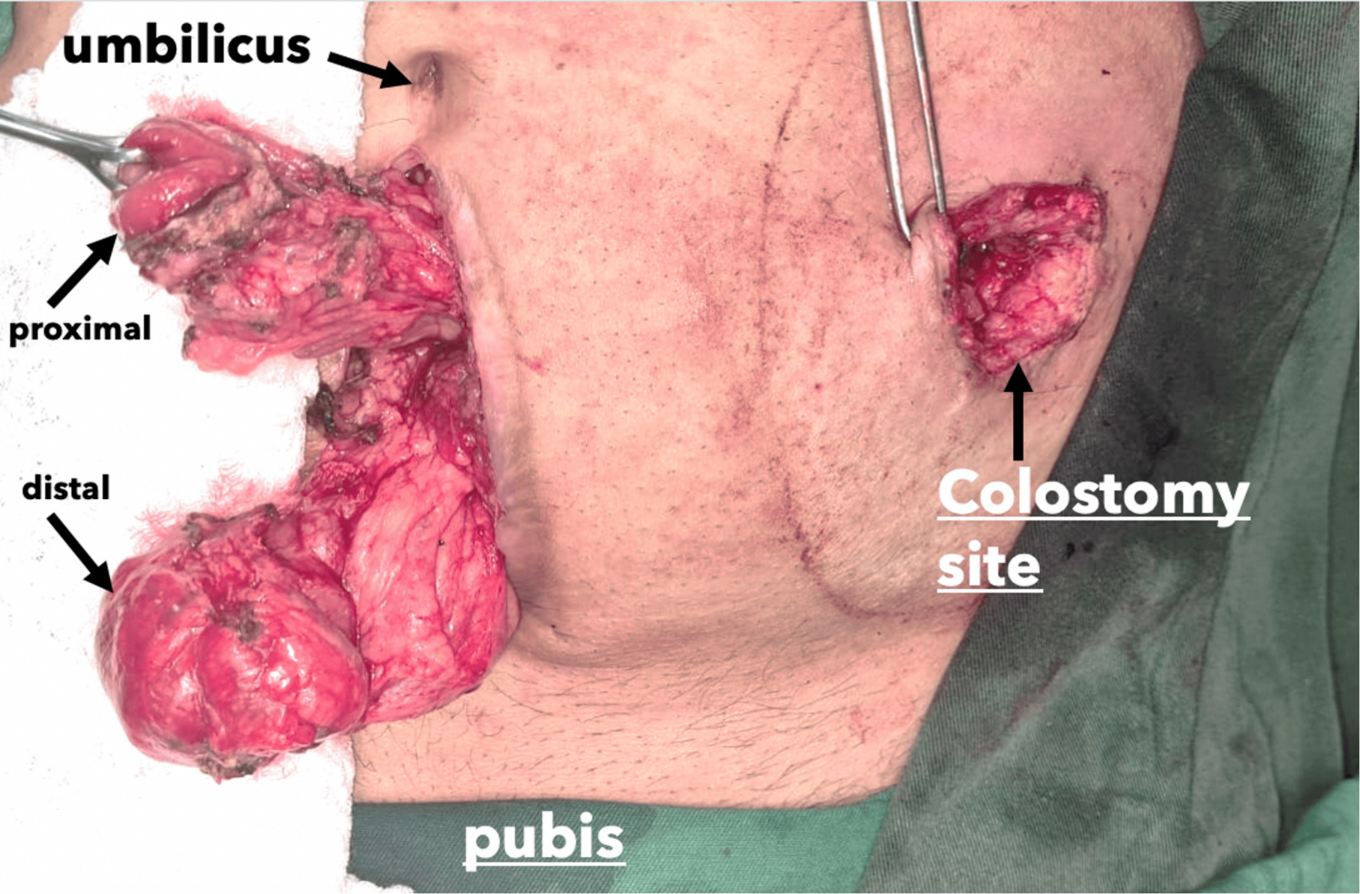
Intraoperative view demonstrating a midline incision during colostomy reversal, with resection of 4 cm of bowel on each side.

**Figure 8:**
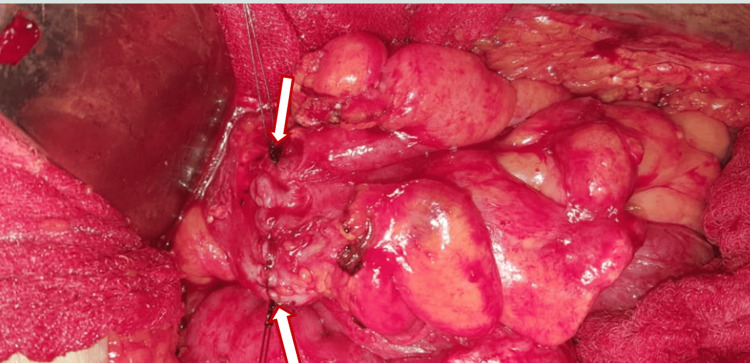
Hand-sewn end-to-end anastomosis of healthy bowel during colostomy reversal following resection of 4 cm of unhealthy bowel. White arrows indicate the start and end of the anastomosis.

The patient was then planned for resection, during which only 4 cm of unhealthy bowel was resected on each side, and the colostomy was reversed with a hand-sewn end-to-end anastomosis (Figures 7-8).

**Figure 9:**
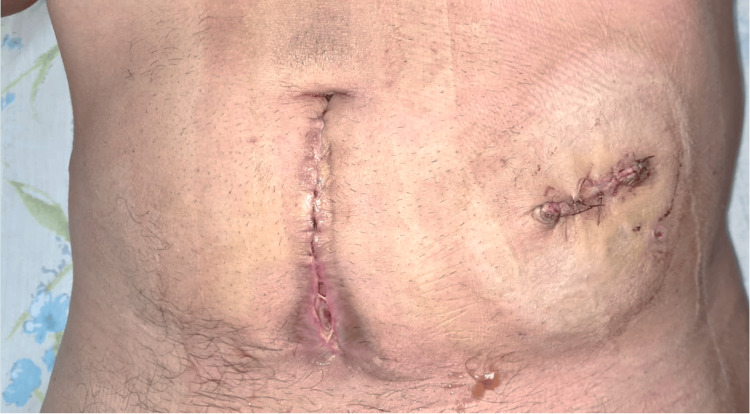
Postoperative view demonstrating a healed midline incision following colostomy reversal, with a healed colostomy closure incision on the right.

Figure 9 shows the healed postoperative incision of the colostomy reversal.

## Discussion

Acute colonic diverticulitis, particularly sigmoid diverticulitis, is among the most common conditions encountered by surgeons in the emergency setting [1]. When complicated by perforation, it typically necessitates urgent surgical exploration. However, the optimal operative strategy for perforated sigmoid diverticulitis has evolved considerably over time. Traditionally, patients with purulent or fecal peritonitis underwent open resection of the affected colon with end colostomy formation (Hartmann's procedure). Although minimally invasive laparoscopic techniques have since been developed and widely adopted, the choice between open and laparoscopic approaches remains complex. This decision must account for patient-specific factors, the surgeon's expertise, and the resources available at the treating institution [2].

Hartmann's procedure was first described in 1921 by Henri Hartmann for the management of rectal cancer, with the aim of reducing operative mortality. By the 1970s, it had gained widespread acceptance for the treatment of perforated diverticulitis. The American Society of Colon and Rectal Surgeons endorsed it as the recommended treatment for Hinchey stages III and IV in 2000. Between 2006 and 2014, there was an emphasis on tailoring management strategies to individual cases. Over the following decade, however, management strategies shifted toward a more individualized approach. By 2020, the Society's guidelines reflected a growing preference for primary resection with anastomosis in appropriately selected patients [4]. Despite these evolving recommendations, the choice of surgical procedure in cases of diffuse peritonitis continues to rely heavily on clinical judgement.

In a comparative study, Constantinides et al. evaluated primary resection and anastomosis - with or without defunctioning stoma - against Hartmann’s procedure in patients with Hinchey stage III-IV diverticulitis. Their findings suggested that in selected patients with diverticular peritonitis, primary anastomosis with proximal diversion may offer an optimal balance between postoperative morbidity, long-term quality of life, and risk of permanent stoma formation [5]. Nevertheless, the stability of this approach depends largely on the condition of the underlying bowel, which remains a critical determinant in selecting minimally invasive and restorative surgical options [6].

Notwithstanding these developments, Hartmann’s procedure continues to play a vital role in the management of generalized peritonitis. It remains a safe and reliable technique for emergency colectomy, particularly in critically ill patients or those with multiple comorbidities [1].

A significant challenge, however, lies in the restoration of bowel continuity following a Hartmann’s procedure. Reversal surgery is associated with considerable morbidity, including wound infection, stoma-related complications, and increased healthcare resource utilisation [7]. The procedure involves resection of the sigmoid colon, closure of the rectal stump, and formation of an end colostomy from the proximal colon. Because the entire sigmoid colon is typically removed, reversal rates remain relatively low [8]. Consequently, a substantial proportion of patients never undergo restoration of bowel continuity and are left with a permanent stoma.

This raises an important question regarding the extent of colonic resection performed during emergency surgery. Traditionally, the degree of resection is determined intraoperatively based on (i) the condition of the bowel, (ii) the extent of intra-abdominal contamination, and (iii) the patient’s hemodynamic stability [9]. Yet, in the acute setting, inflammatory edema of the mesentery and peritoneum can exaggerate the apparent severity and distribution of the disease, potentially leading to more extensive resection than necessary.

In this present case, the degree of abdominal sepsis precluded safe resection with primary anastomosis at the initial operation. Instead, the sigmoid colon was divided at the site of perforation. The proximal end was exteriorized as an end colostomy, while the distal segment was oversewn and secured to the anterior abdominal wall near the colostomy site. Notably, no formal resection of the sigmoid was performed. This approach allowed for control of the septic focus without committing the patient to extensive resection in a hostile inflammatory environment.

Addressing the immediate septic crisis while deferring definitive resection may offer several advantages. During the acute inflammatory phase, edema and tissue friability increase the risk of complications such as anastomotic leakage and surgical site infection. Allowing the inflammatory process to resolve before undertaking definitive resection may reduce these risks. Moreover, although diverticulitis can result in scarring, thickening, or luminal narrowing, the bowel adjacent to the perforation may remain relatively healthy and amenable to more limited resection once inflammation subsides [8].

Delaying resection also permits a more thorough evaluation of the colon in a stable, elective setting. With careful preoperative planning and optimization of the patient’s condition, subsequent surgery can be more targeted and conservative. A limited resection performed months later, if indicated, may preserve a greater length of healthy colon both proximally and distally, potentially improving functional outcomes and reducing long-term morbidity.

In summary, while Hartmann’s procedure remains an essential option in the emergency management of perforated diverticulitis, selective nonresection at the index operation may represent a viable alternative in carefully chosen cases. By prioritizing sepsis control and deferring definitive resection, surgeons may reduce unnecessary colonic sacrifice and improve the likelihood of eventual restoration of bowel continuity.

## Conclusions

It is evident that the optimal management of perforated sigmoid diverticulitis has evolved significantly. While traditional approaches like Hartmann's procedure have been widely used, advancements in less invasive techniques offer promising alternatives. By managing the acute phase conservatively and delaying resection until the inflammation subsides, there is potential to reduce postoperative complications and improve patient outcomes. Moreover, the decision-making process must consider individual patient factors, available resources, and surgical expertise, particularly in diverse settings like the Caribbean. Continued research and tailored strategies are essential for refining treatment protocols and ensuring the best possible care for patients with this condition.
